# Evaluation of the efficacy, safety, and stability of posterior chamber phakic intraocular lenses for correcting intractable myopic anisometropic amblyopia in a pediatric cohort

**DOI:** 10.1186/s12886-021-02074-3

**Published:** 2021-08-28

**Authors:** Fathy Fawzy Morkos, Nader F. Fawzy, Mohamed El Bahrawy, Nada Fathy, Rania Serag Elkitkat

**Affiliations:** 1 Watany Research and Development Center, Watany Eye Hospital, Cairo, Egypt; 2 Sehkraft Augenzentrum, Cologne, Germany; 3 Faculty of Medicine, Ain Shams University, Cairo, 11799 Egypt

**Keywords:** Pediatric ICL, Posterior chamber phakic IOL, Visian ICL, Anisometropic amblyopia, Pediatric ICL outcomes, Prevention of amblyopia

## Abstract

**Background:**

Myopic anisometropic amblyopia in pediatrics is one of the most challenging clinical situations that can face an ophthalmologist. Conventional correction modalities for myopic anisometropia, using spectacles, contact lenses, and/or occlusion therapy, may not be suitable for some pediatric patients or for some ocular conditions. This may lead to the development of anisometropic amblyopia. The aim of the present study was to evaluate the visual and the refractive efficacy, safety, and stability of Posterior Chamber Phakic Intraocular Lenses (PC-pIOLs) for correcting myopic anisometropic amblyopia in a pediatric cohort.

**Methods:**

This case series, prospective, interventional study was conducted at Watany Eye Hospital, Cairo, Egypt. It comprised children and teenagers with myopic anisometropic amblyopia and unsuccessful conventional therapy. After implantation of Intraocular Collamer Lenses “ICLs” (Visian ICL, Model V4c, STAAR Surgical, Monrovia, California, USA), postoperative follow-up visits were scheduled, with automated refraction and Pentacam imaging performed.

**Results:**

The study enrolled 42 eyes of 42 patients. The age range was 3 to 18 years (mean ± SD = 10.74 years ±4.16). The mean preoperative spherical equivalent (SE) was − 12.85 D ± 2.74. The results declared a significant improvement in the postoperative Corrected Distance Visual Acuity “CDVA” (*P* value < 0.01) and SE (*P* value < 0.01). The efficacy index had a value of 1.18 ± 0.3 and the safety index was 1.09 ± 0.24. The follow-up visits had a mean ± SD of 14.67 months ±16.56 (range of 1 to 54 months). The results showed a refractive stability, with statistically insignificant improvements in the patients’ visual acuity and refractive status on evaluating the enrolled pediatrics during the follow-up visits compared to the first postoperative visits. No postoperative complications were encountered. Worthy of mention is that there was a significant (80%) non-compliance with the prescribed postoperative occlusion therapy.

**Conclusions:**

The present study, with the longest reported follow-up range, declared the long-term efficacy, safety, and stability of Visian ICLs for correcting myopic anisometropic amblyopia in pediatrics. The reported non-compliance with occlusion therapy validates the early implantation of Visian ICLs in cases with failed conventional therapy to guard against anisometropic amblyopia.

## Background

Amblyopia development in pediatric patients is one of the most challenging situations that can face an ophthalmologist. Its prevention and correction require proper cooperation of the child and his/her guardians, which is difficult to achieve in many instances [1]. Anisometropic amblyopia is a common amblyopic form which leads to aniseikonia and unilateral image blur, with a consequent suppression of this blurred image by the brain [2].

The conventional correction of anisometropia using spectacles remains the gold standard that is adopted by many pediatric ophthalmologists. Children can, in many instances, tolerate glasses while having large refractive differences between both eyes. This is mainly encountered with axial rather than refractive myopia, assuming Knapps’ law of visual optics. However, literature has demonstrated an induced stretching of the retina with significantly long globes that can be a primary cause of reduced spatial resolution in the peripheral field [3]. This renders the glasses an inconvenient corrective modality in a major portion of myopes with high errors or those having refractive rather than axial myopia. Besides, if anisometropic amblyopia develops, occlusion or penalization of the fellow eye can be challenging and difficult to implement [4]. Contact lenses (CLs) are another available option for correcting anisometropia. Nonetheless, intolerance to their use and poor compliance, especially with younger age groups, can lead to treatment failure [5].

When spectacles and CLs fail to guarantee the desired visual acuity for the pediatric age group, other treatment modalities should be addressed to prevent amblyopia. Corneal excimer laser ablative procedures are an available alternative. Yet, the risks of flap related complications, postoperative corneal haze, and the possible development of corneal ectasia are higher among the pediatric patients [6–8]. Another refractive correction modality for amblyopia with higher values of refractive errors is refractive lens exchange. Such procedures however carry major disadvantages, the most prominent of which are the greater risks of retinal detachments and the permanent loss of the accommodative power [9].

The use of phakic intraocular lenses (pIOLs) has been proposed as an effective modality for correcting intractable anisometropic amblyopia in children [10]. The major advantages of using pIOLs include their predictability, high optical quality, preservation of the child’s accommodative power, and avoiding the hazards of corneal ablative procedures [11–13]. Though previous studies reported that both iris-fixated pIOLs and posterior chamber pIOLs (PC-pIOLs) have equally satisfactory postoperative visual outcomes, implantation of iris-fixated pIOLs carries a higher risk of endothelial cell loss and intraocular inflammation in adulthood. On the other side, PC-pIOLs have a significantly lower risk of such complications [10]. Though the implantation of a PC-pIOL in a child seems more convenient than an iris fixated pIOL, it can induce other complications that usually arise from preoperative miscalculations or mispositioning of the IOL, including mainly anterior subcapsular cataract formation and shallow anterior chamber [14]. Though reports of such complications in the pediatric population are few, this may be attributed to the paucity of studies on this age range, especially for long follow-up intervals [10].

The aim of the present study was to evaluate the refractive efficacy, safety, and stability of PC-pIOLs (Visian Intraocular Collamer Lenses “ICLs”) in a pediatric cohort with myopic anisometropic amblyopia. The primary outcome was to assess the visual performance of the enrolled pediatrics 1 month following the surgical intervention and at their last follow-up visit, while the secondary outcomes were to detect the long-term stability and the possible long-term complications, by performing slit lamp examination, IOP measurement, and Pentacam examination.

## Patients and methods

This is a prospective, consecutive, non-controlled, interventional, case series study that was performed on a pediatric group of patients (aged 3 to 18 years) who sought medical advice at Watany Eye Hospital, Cairo, Egypt. All the recruited patients performed the surgical procedure in the period from January 2016 to July 2020. The study adhered to the tenets of the Declaration of Helsinki and was conducted in compliance with the Ethical Standards set by the Institutional Review Board of the Watany Research and Development Center (the registration code is REF-2016-002). The guardians of the participating children and teenagers signed preoperative informed consents and were counselled about the nature of the surgical technique and the possible postoperative outcomes.

The exclusion criteria included pediatric patients with previous ocular trauma or surgeries, corneal pathologies (mainly corneal dystrophies or ectatic conditions), angle anomalies, congenital glaucoma, any lenticular abnormalities [including abnormal lenticular shapes (mainly spherophakia and microspherophakia), abnormal lens positions (ectopia lentis), and lenses with cataractous changes], Anterior Chamber Depth (ACD) less than 2.8 mm, and any posterior segment abnormalities. Besides, cases with high cylindrical errors (either exceeding 3 D in the operated eye or having a difference in the cylindrical component between both eyes of more than 2 D) were excluded from the selected candidates. Hirshberg and cover tests were performed during the patients’ clinical examination to evaluate the existence of strabismus, where any participant with co-existing manifest strabismus was excluded from the study and was referred to a strabismus consultant for proper detailed evaluation and re-assessing the proper management thereafter.

The study enrolled children and young teenagers with myopic anisometropic amblyopia (errors of range − 6 to − 18 Diopters “D” were included) and unsuccessful conventional amblyopic therapy (using spectacles, contact lenses, and/or occlusion therapy). Other than analyzing the results for the whole pediatric cohort, two subgroupings were performed for the enrolled participants based on both age and refractive condition of the other eye. For the age subgrouping, the participants were subdivided into three groups; group 1 (aged 3 to 6 years), group 2 (aged 7 to 12 years), and group 3 (aged 13 to 18 years). As regards to the subgrouping based on the refractive condition of the other eye, group 1 included pediatric patients with low myopia (more than 1 D and less than 6 D), while group 2 comprised patients with myopia of less than 1 D or emmetropia. Both groups were compared regarding the visual performance (Unaided Distance Visual Acuity “UDVA”, Corrected Distance Visual Acuity “CDVA”, and Spherical Equivalent “SE”) of the eye with the pediatric ICL implantation.

For all candidates of the case series, a baseline ophthalmological examination was performed before the surgical intervention. This included automated refraction for measuring the SE (which was performed under complete cycloplegia with cyclopentolate 1%) and assessment of UDVA and CDVA. Subjective refraction was tried with clinical judgement which relied mainly on the refraction obtained from the automated autorefractometer. Snellen acuity chart was conventionally used, but for the very young children where the Snellen acuity was inconvenient, Sheridan-Gardiner test was used. No crowding was used on subjective visual assessment. The visual acuity was then converted to LogMAR for the statistical analysis. Furthermore, slit lamp examination, intraocular pressure (IOP) measurement using air puff tonometer, and fundus examination by indirect ophthalmoscopy were done for all participants. Hirschberg and cover tests were performed to reassure orthophoria and patients with existing strabismus were excluded.

Prior to the selection of the suitable candidates, good quality scans of the Pentacam HR, branded as Allegro Oculyzer II (WaveLight, Erlangen, Germany, software version 1.20r20) were captured for all the patients to rule out corneal ectatic conditions and to measure the ICL vault. Essential biometric measurements were performed, including the ACD, central corneal thickness, keratometric values, and measuring the white-to white (WTW) diameter. To ensure the proper sizing of the ICL and for calculation of its suitable power as well as validation of the WTW and ACD measurements, the values obtained from the Pentacam HR were also confirmed by capturing good quality scans from the IOL Master 500 (Carl Zeiss Meditec, Germany) for all the enrolled participants. The ACD values in all patients exceeded 2.8 mm, which was measured from the anterior lens surface to the corneal endothelium.

Statistical comparisons were made between the preoperative parameters and the corresponding ones in the two postoperative visits. Furthermore, plotting of Pearson correlations was performed to determine the possible relations between the visual improvements (in UDVA, CDVA, and SE) and the major variables that were assumed to possibly affect it (namely patients’ age and the difference in refraction between both eyes). The results of these possible relations were furtherly validated by including the most significant contributors in regression models (univariate and multivariate analyses) to estimate their effects on the outcomes.

### Surgical technique

The surgical technique was performed for all the cases by the same experienced surgeon (F.F.M). All the surgeries were carried out under general anesthesia due to the young age group. All the pediatric participants implanted a Visian ICL (Model V4c, STAAR Surgical, Monrovia, California, USA), which was introduced and positioned into its proper location as per the conventional method of its implantation [15]. On entering the patients’ refractive powers in the online calculation software of the company, emmetropia was targeted for the recruited participants.

### Postoperative management

Postoperatively, eyedrops containing a steroid/antibiotic combination were prescribed 4 times daily and tapered out weekly for 1 month. The suture was removed 2 weeks after surgery. Automated refraction and subjective visual assessment (using Snellen acuity chart or Sheridan-Gardiner test in younger ages), slit lamp examination, IOP measurement using air puff tonometry, and Pentacam HR were performed for all the participating pediatrics along the follow-up visits, where the first visit was scheduled to be 1 month after the surgical intervention, and the data from the last follow-up visit for each participant was enrolled in the study. All the aforementioned examinations were done for all the participants on both postoperative visits, except for the Pentacam evaluation that was only performed on the last follow-up visit. The Pentacam images were evaluated to document the ICL stability, the value of the anterior ICL vault, and any detectable postoperative complications. Occlusion of the fellow eye during the day was prescribed for at least 3 h, combined with 1 hour of near visual activities [16], along the first month following surgery. The occlusion therapy was prescribed thereafter if necessary. Adherence to the prescribed occlusion was assessed during the patients’ follow-ups by reporting the detailed way of performing it.

### Statistical analysis

Data analysis was performed using IBM SPSS Statistics for Windows (Version 25.0. Armonk, NY: IBM Corp.). The one-sample Kolmogorov-Smirnov test was used to test for normality. Quantitative data were presented as mean, standard deviation (SD) and ranges. Sex differences were evaluated by the chi-squared test. The comparison between more than two paired groups with quantitative data and non-parametric distribution was done by using Kruskall Wallis Test followed by post hoc analysis using Wilcoxon Rank test. Pearson correlation coefficients were used followed by univariate and multivariate linear regression using enter method to assess the correlation between different variables and the improvement of visual parameters. The confidence interval was set to 95% and the margin of error accepted was set to 5%. So, the *p*-value was considered significant at the level of < 0.05. Both the efficacy and the safety indices for the ICL implantation were calculated for the recruited cohort, where the cut-off level of the efficacy index was set to 0.80 and that of the safety index was set to 0.85.

## Results

The present study was conducted on 42 eyes of 42 children with unilateral high myopia or myopic anisometropic amblyopia, where the ICL was implanted in the more ametropic eye. The age range of the recruited pediatric cohort was 3 to 18 years, with a mean ± SD of 10.74 years ±4.16. The female to male percentage was 40.5 to 59.5%. Twenty-two eyes were right while 20 eyes were left. The mean preoperative SE was − 12.85 D ± 2.74 (range of − 19.00: − 7.13 D), the mean preoperative cylindrical error was − 2.17 D ± 1.05, while the mean Visian ICL power was − 12.77 D ± 2.39 (range of − 18.00: − 9.00 D). The follow-up visits had a mean ± SD of 14.67 months ±16.56 (range of 1 to 54 months). Since many patients were non-compliant with the regular follow-up intervals that were pre-set before the study (either due to the COVID-19 circumstances or due to living in remote governorates), only the data from the first follow-up visit (1 month after surgery) and from the last fulfilled follow-up (considered as the second visit) was enrolled in the study.

Table 1 shows the mean values of the patients’ visual acuity and refraction on each of the preoperative visit, the first postoperative visit, and the last follow-up visit and the *P*-values of significance between them. The results declared statistically significant differences between the values of the preoperative and the first postoperative visit, with a significant improvement in each of the postoperative UDVA (*P* value < 0.01), CDVA (*P* value < 0.01, with a mean improvement of 0.2 LogMAR ±0.50), and SE (*P* value < 0.01, with mean improvement of − 11.83 D ± 4.78). Moreover, the results obviously showed refractive stability among the participating patients, as there was a slight (statistically insignificant) improvement in all the mean values of the patients’ visual acuity and refraction between the first postoperative and the last follow-up visit, except for a single statistically significant improvement in the UDVA (*P* value =0.012). The values of both the efficacy and the safety indices for the enrolled patients were determined and showed remarkably high values of 1.18 ± 0.3 and 1.09 ± 0.24, respectively.

**Table 1:** Statistical comparisons between the mean values, standard deviations, and ranges of the patients’ visual acuity and refraction on each of the preoperative visit, the first postoperative visit, and the last follow-up visit

		Preoperative visit	1st postoperative visit	Last postoperative visit	Test value	***P***-value of significance	Significance level
**UDVA**^**b**^	Mean ± SD^b^	1.33 ± 0.53	0.57 ± 0.36	0.42 ± 0.34	30.481825^a^	**< 0.001**	HS^b^
	Range	0.04–2	0–1.52	0–1.3			
**CDVA**^**b**^	Mean ± SD	1.06 ± 0.56	0.42 ± 0.35	0.21 ± 0.18	14.880825^a^	**0.001**	HS^b^
	Range	0.18–2	0.04–1.52	0–0.7			
	Range	−17.5 – −6	−6.25 – 2.5	− 1.75 – 4.75			
	Range	−4.5 – 0	−50 – 1.5	−4.5 – 1.5			
**SE**^**b**^	Mean ± SD	−12.85 ± 2.74	− 1.02 ± 3.97	−0.55 ± 1.45	48.825^a^	**< 0.001**	HS^b^
	Range	− 19 – −7.13	−24 – 1.63	− 2.63 – 4.13			
**Post hoc analysis**							
		**Preoperative Versus 1st postoperative visit**	**Preoperative Versus Last postoperative visit**		**1st postoperative Versus Last postoperative visit**		
**UDVA**^**b**^		**< 0.001**	**< 0.001**		**0.012**		
**CDVA**^**b**^		**< 0.001**	**0.001**		0.125		
**SE**^**b**^		**< 0.001**	**< 0.001**		0.588		

*P*-value > 0.05: Non significant; *P*-value < 0.05: Significant; *P*-value < 0.01: Highly significant

^a^Friedman test followed by post hoc analysis using Wilcoxon Rank test

^b^*UDVA* Unaided Distance Visual Acuity, *SD* Standard Deviation, *CDVA* Corrected Distance Visual Acuity, *SE* Spherical Equivalent, *HS* Highly significant

The slit lamp examination during the first postoperative and the last follow-up visit showed clear corneas, quiet anterior chamber (AC) with no detected inflammatory reactions or pigmentary deposits, and a centralized ICL. The Pentacam images which were captured on the last follow-up visit confirmed the stability of the ICL in place, with no detected obstruction of the AC angle, and a sufficient space between the ICL and the crystalline lens. The anterior ICL vaulting had a mean and SD of 490 um ± 40.23.

As regards to the IOP measurements and the fundus examination of the participants, the findings were unremarkable before and after the surgical performance and during the follow-up visits.

A significant portion of the children’s guardians (80%) reported poor compliance with the prescribed occlusion therapy, despite strict instructions that were given to abide by it. Yet, the parents of all the children reported enhanced physical activities and improved social intermingling for all the participating patients within a short time interval of performing the surgical intervention.

Regarding the age subgrouping, our examined cohort included 7 patients in group 1, 18 patients in group 2, and 17 patients in group 3. No significant differences were detected among the three age subgroups regarding the visual or refractive changes before and after the surgical procedure. For the subgrouping that was based on the refractive status of the fellow eye, group 1 patients had a spherical equivalent that ranged between − 1.25 and − 4.75 D, and the results declared no statistically significant differences between the patients of group 1 (17 eyes) and group 2 (25 eyes) regarding the visual performance of the eye that performed the pediatric ICL implantation.

The results of the performed Pearson correlations showed a single significant relation, where the difference in refraction between both eyes was negatively correlated with the improvement of SE (*r* = − 0.83, *P* value < 0.001). This relation was furtherly augmented by the linear regressions, with the same significant relation detected in both the univariate (beta coefficient = − 0.73, *P* value < 0.001) and the multivariate (beta coefficient = − 0.83, *P* value < 0.001) analyses. Contrarily, the age factor was not correlated with any of the included parameters.

## Discussion

This prospective case series study showed the efficacy (efficacy index value of 1.18 ± 0.3), safety (safety index value of 1.09 ± 0.24), and stability of Visian ICLs for correcting myopic anisometropic amblyopia in a pediatric cohort with unilateral high myopia and non-compliance with the conventional treatment modalities. To date, the present study comprised the largest number of pediatric patients who implanted an ICL for correcting anisometropic amblyopia, and it is also the first study to document this long follow-up interval that reached up to 54 months. Thus, the present report validates the use of Visian ICLs in young children and teenagers without concerns about their long-term refractive stability or about the development of long-term complications. Besides, the absence of significant differences in the visual performance among the three age subgroups indicates promising results for the whole included pediatric age range (3 to 18 years).

Our studied population included cases of unilateral high myopia. This population was shown to be more prone to develop anisometropic amblyopia, even with trials of conventional treatments using spectacles, contact lenses, and occlusion therapy [17].

For all candidates included in the present study, the cylindrical component did not exceed 3 D in the operated eye and the difference in the cylindrical component between both eyes was no more than 2 D. We excluded patients having higher cylindrical errors that would require toric ICLs for correcting this high astigmatism, assuming that the corneal toricity will change along the time and thus implanting a toric ICL at this young age would possibly require a secondary exchange within few years. Even though our enrolled patients had relatively low cylindrical values, the patients who were left postoperatively with a visually-significant cylinder were corrected by spectacles, especially that this study aimed at correcting the anisometropic amblyopia rather than attaining glass independence for the candidates.

Implantation of PC-pIOLs in children for preventing and treating anisometropic amblyopia can be considered as a preferable technique by many surgeons, and also, after proper counselling, by many parents. This can be attributed to the efficacy and safety of the procedure in restoring the visual performance, the lack of post-operative noxious precautions which are encountered with the corneal refractive surgeries (especially with the younger ages), the significantly lower risk of endothelial cell loss than the AC-IOLs (especially with the inevitable eye rubbing in children), the unbreaching of the corneal architecture (allowing for future successful corneal refractive surgeries if needed), and the reversible nature of the technique (if needed) [18].

The enrolled pediatric cohort in the present study did not experience post-operative complications. Lack of surgical experience and an improper vault size are the two main reported risk factors for a higher incidence of developing pediatric secondary cataract (in cases with low vaults) or pupillary block glaucoma (with high vault values) following the surgical intervention [18]. Yet, it is noteworthy that the complications related to the improper ICL vault were more frequently encountered with the older ICL models. The newer model ICLs (V4c used in this study as well as the newer model V5) include a central port which greatly minimizes the risk of either cataract development or pupillary block [19]. The absence of the two aforementioned risk factors in our study may clearly explain the absence of post-operative complications in our recruited patients.

In our studied pediatric population, the mean vault value was within the normal ranges and towards the higher normal values. A relatively higher value for the pediatric ICL vault has been advocated, considering the expected progressive reduction of the central vault over time with the slow (yet steady) axial growth of the crystalline lens over the years. That is why higher vault values (within the normal ranges) can be more preferable for younger age groups [20].

Worthy of mention is that the compliance with the occlusion therapy was poor for most of the pediatric cohort, which has also been reported in previous studies [21, 22]. This can be attributed to many factors, including mainly skin irritation, poor cosmetic appearance, lengthy treatment periods, and the stress suffered by the child and his parents. These factors make the occlusion therapy difficult to achieve and more likely to be abandoned or applied considerably less than required. This validates the use of the ICLs at an early phase if the conventional therapy is ineffective, so as to avoid the occurrence of anisometropic amblyopia.

The parents of the pediatric cohort reported improved physical and social activities within a short period of the ICL implantation. These short-term enhancements cannot be attributed to simple maturation of the children that requires longer time intervals, so we can attribute these improvements to the better visual performance following the ICL implantation.

Previous studies reported the outcomes of implanting iris-fixated ICLs for correcting pediatric anisometropic amblyopia. Though the visual outcomes were satisfactory, some complications were documented, including progressive endothelial cell loss with eye rubbing (that is mostly uncontrollable with younger ages) and iris chaffing. Furthermore, the relatively short follow-up intervals render the results of these studies unreliable for the true evaluation of the possible consequent complications [20, 23–28].

To the authors’ knowledge, few case series studies were conducted on implanting PC-pICLs for myopic anisometropic children. All these studies recruited a fewer number of children than the present study, and the follow-up ranges were shorter. Table 2 displays the clinically relevant results of these studies, which are collectively in accordance with our study results in validating the stability and the absence of significant complications after implanting PC-pICLs [29–33].

**Table 2:** Summary of the clinically-relevant data of the previous studies performed on the implantation of Posterior Chamber Visian Intraocular Collamer Lenses (ICL) for pediatric cohorts

Authors	Year	Number of eyes	Range (mean) of age (years)	Mean Preoperative Unaided Distance Visual Acuity	Mean Preoperative Corrected Distance Visual Acuity	Mean Preoperative Spherical Equivalent	Mean ICL power	Mean Post-operative Unaided Distance Visual Acuity	Mean Post-operative Corrected Distance Visual Acuity	Mean Post-operative Spherical Equivalent	Follow Up Period (months)
BenEzra et al.	2000	3	Reported as individual cases (9,14, and 18 years old), and all showed improvements in the visual performance.								
Elmassry et al.	2017	12	2–15 (8)	0.9	0.4	−11.28	–	0.0.3	0.1	− 1.82	12
Lesueur and Arne	2002	5	3–16 (9)	–	–	−12.8	−15.5	All gained lines	All gained lines	+ 0.5	11.8
Alio et al.	2011	11	2–15 (8)	1.1	0.84	−10.14	–	0.65	0.36	−1.16	60
TYCHSEN et al.	2017	40	2–17 (10)	1.72	–	−9.2	–	0.48	–	+ 0.56	15.1
Elmassry et al.	2017	12	2–15 (8)	0.9	0.4	−11.28	–	0.0.3	0.1	−1.82	12
BenEzra et al.	2000	3	Reported as individual cases (9,14, and 18 years old), and all showed improvements in the visual performance.								

In our study, the age subgrouping detected equivocal visual results, and the performed linear regression analysis did not show a significant relation between the visual improvement and the age factor, denoting that the ICL implantation in such a cohort is a preferable technique for all the included age range. Although the age subgrouping of our enrolled pediatrics yielded no significant differences among the three subgroups, future studies conducted on larger cohorts are needed to validate these results, especially that the number of patients in the smallest age subgroup (aged 3 to 6) was smaller than the other two subgroups.

We also performed a subgrouping for the enrolled cohort that was based on the refractive condition of the fellow eye. This aimed to declare whether the eyes with low myopia in the fellow eye had a more favorable visual prognosis along the follow-up visits than those with emmetropia in the fellow eye, assuming that the amblyopic eye will be favored from the refractive aspect after the ICL implantation. Although our study did not show significant differences between the two subgroups, we believe that these results should be negated or reinforced by future studies performed on larger cohorts and having a more equivocal number of patients in both groups (as the eyes in group 1 with low myopia in the fellow eye represented 39.5% only of the enrolled patients).

In our studied cohort, specular microscopy was not performed for the patients, as we did not expect a significant compromise for the corneal endothelium by the implanted ICLs (owing to their posterior location behind the iris). Previous reports have documented that PC-pICLs are much safer on the corneal endothelium than AC-IOLs [18]. Moreover, a recent study by Fernández-Vega-Cueto and his co-workers [34] reassured the lack of long term traumatizing effects of the modern ICL designs on the corneal endothelium, where their study showed a very minimal endothelial cell loss of 2.6% after V4c ICL implantation at the last follow-up visit along a follow-up interval of 7 years. Future studies along longer follow-up periods can more robustly declare the refractive stability and the safety of the Visian ICLs. Besides, highlighting the impact of improving the visual performance on the binocular vision is recommended in the upcoming studies.

## Conclusions

In conclusion, the present study declared the long term visual and refractive efficacy, safety, and stability of the Visian ICL for correcting myopic anisometropic amblyopia in a pediatric cohort with a mean preoperative SE of 12.85 D ± 2.74 and a range of − 19.00 to − 7.00 D. Based on our study results, the implantation of Visian ICLs for cases of unilateral high myopia with intractable anisometropic amblyopia results in a long-term visual and refractive stability, and can also be gauged as a low risk procedure, evidenced by the long-term absence of reported complications. Furthermore, the reported non-compliance with occlusion therapy in many of our studied pediatrics validates the early implantation of Visian ICLs in cases of failure of the conventional conservative correction and occlusion therapy to guard against anisometropic amblyopia.

## Data Availability

The datasets used and/or analysed during the current study are available from the corresponding author on reasonable request.

## Abbreviations

CLs: Contact lenses
pIOLs: phakic intraocular lenses
PC-pIOLs: Posterior chamber pIOLs
ICLs: Intraocular Collamer Lenses
ACD: Anterior Chamber Depth
D: Diopter
UDVA: Unaided Distance Visual Acuity
CDVA: Corrected Distance Visual Acuity
SE: Spherical Equivalent
IOP: Intraocular pressure
WTW: White-to white
SD: Standard deviation
AC: Anterior chamber
